# Low prevalence of *GCK* gene mutations in Chinese patients with gestational diabetes mellitus

**DOI:** 10.1016/j.clinsp.2026.100889

**Published:** 2026-02-25

**Authors:** Zhixin Wang, Lili Huo, Ling Lan, Yongzeng Chen, Qingyao Zuo, Wei Deng

**Affiliations:** a Department of Endocrinology and Metabolism, Beijing Jishuitan Hospital, Capital Medical University, Beijing, China; b Department of Cardiology and Macrovascular Disease, Beijing Tiantan Hospital, Capital Medical University, Beijing, China

**Keywords:** *GCK* Gene, Chinese, Gestational diabetes mellitus, Maturity onset diabetes of the young (MODY)

## Abstract

**Background and aim:**

Accumulating evidence suggests maturity onset diabetes of the young (MODY) caused by *GCK* gene mutations (*GCK*-MODY) is often misdiagnosed as Gestational Diabetes Mellitus (GDM) in pregnant women. This study investigated the prevalence and diagnostic value of *GCK* gene mutations in Chinese GDM patients.

**Methods:**

A retrospective analysis was conducted on 3394 pregnant women undergoing prenatal care or delivery at Beijing Jishuitan Hospital between April and December 2019. GDM was diagnosed in 474 women (14.0%) using the International Association of Diabetes and Pregnancy Study Groups (IADPSG, 2010) criteria via a 75 g Oral Glucose Tolerance Test (OGTT). Among these, 177 GDM patients with fasting blood glucose ≥5.1 mmoL/L underwent targeted *GCK* gene sequencing.

**Results:**

Targeted sequencing identified eight rare *GCK* variants in nine individuals. None of the variant carriers met definitive diagnostic criteria for *GCK*-MODY. The prevalence of rare *GCK* variants in the screened GDM subgroup was 1.9% (9/474).

**Conclusion:**

The low prevalence (1.9%) of rare *GCK* variants and absence of confirmed *GCK*-MODY cases indicate limited utility of fasting glucose-based screening for *GCK*-MODY in Chinese GDM populations. These findings highlight challenges in distinguishing *GCK*-MODY from GDM through conventional glucose thresholds.

## Highlights

• Screened 177 Chinese GDM women (FBG ≥5.1 mmoL/L) for GCK variants.
• Identified 8 rare GCK variants in 9 individuals (1.9 % prevalence).
• No confirmed GCK-MODY cases found among variant carriers.
• Limited utility of FBG-based GCK-MODY screening in Chinese GDM.

## Introduction

The *GCK* gene encodes glucokinase, a key enzyme involved in Glucose-Stimulated Insulin Secretion (GSIS). Heterozygous loss-of-function mutations in the *GCK* gene result in Maturity-Onset Diabetes of the Young (*GCK*-MODY), a common form of monogenic diabetes.^1^ Unlike type 1 and type 2 diabetes, patients with *GCK*-MODY typically do not require pharmacological treatment and have a relatively favorable prognosis.^2^ Additionally, as an autosomal dominant genetic disorder, the blood glucose management strategy during pregnancy for *GCK*-MODY patients is more dependent on maternal and fetal genotypes than on maternal blood glucose levels.^3^ Therefore, identifying *GCK*-MODY, especially in populations with Gestational Diabetes Mellitus (GDM), is crucial for ensuring appropriate management. However, *GCK*-MODY is often difficult to diagnose clinically due to the absence of typical diabetic symptoms (e.g., polyuria, polydipsia) and a lack of specific diagnostic markers, and genetic testing is often required for confirmation.^2^

*GCK*-MODY patients are frequently misdiagnosed with GDM due to elevated blood glucose detected during pregnancy. Studies have shown that *GCK*-MODY can be distinguished from GDM using specific screening methods. However, differences in ethnicity, screening methods, and diagnostic criteria contribute to significant variability in the reported prevalence of *GCK*-MODY among GDM populations^4^, ^5^, ^6^, ^7^, ^8^, ^9^, ^10^, ^11^, ^12^, ^13^, ^14^, ^15^, ^16^, ^17^, ^18^ (Table 1). Reliable and cost-effective screening methods for *GCK*-MODY remain unavailable.^17^^,^^19^ This study aims to evaluate the prevalence of *GCK*-MODY among Chinese pregnant women and to explore suitable screening criteria.

**Table 1:** Prevalence of *GCK*-MODY in GDM across different populations and screening criteria.

Authors	Country (Ethnicity)	Screening Criteria	Prevalence of *GCK*-MODY in Tested GDM Group (n)	Prevalence of *GCK*-MODY in Total GDM Population (n)
Stoffel et al.^4^	American (Multi-ethnic)	GDM with a first-degree relative with diabetes	5 % (2/40)	UNK
Zouali et al.^5^	French	History of GDM and family history of non-insulin-dependent diabetes mellitus	6 % (1/17)	UNK
Saker et al.^6^	UK (Caucasian)	GDM with postpartum persistent hyperglycemia (5.5–10.0 mmoL/L)	6 % (3/50)	UNK
Ellard et al.^7^	UK (Caucasian)	Four criteria: 1) Persistent fasting hyperglycemia outside pregnancy (5.5–8.0 mmoL/L); 2) Small increment (< 4.6 mmoL/L) during 2 h OGTT; 3) Insulin treatment during pregnancy but controlled on diet; 4) Family history of T2DM, GDM, or fasting hyperglycemia (>5.5 mmoL/L) in a first-degree relative.	80 % (12/15)	UNK
Kousta et al.^8^	UK (Multiethnic)	Fasting hyperglycemia (5.5–8.0 mmoL/L) in pregnancy and outside pregnancy; glucose increment < 3.5 mmoL/L during 75 g OGTT postpartum.	12 % (2/17)	UNK
Zurawek et al.^9^	Polish	Age < 35-years, BMI before pregnancy < 25 kg/m^2^, glucose increment < 4.6 mmoL/L during 2 h OGTT, and family history of T2DM or GDM in a first-degree relative.	2 % (3/119)	UNK
Lukasova et al.^10^	Czech	Diagnosis of GDM	0 % (0/141)	0 %
Frigeri et al.^11^	Brazil	Diagnosis of GDM	0 % (0/100)	0 %
Chakera et al.^12^	UK and Ireland (Anglo-Celtic)	Fasting blood glucose ≥5.1 mmoL/L in OGTT	1.0 % (4/390)	0.9 % (4/447)
Rudland et al.^13^	Australia (Multiethnic)	Combined criteria: BMI < 25 kg/m^2^ and fasting glucose ≥ 5.5 mmoL/L	12.9 % (4/31)	0.5–1 %
Gjesing et al.^14^	Danish	Diagnosis of GDM and treated with diet	1.9 % (7/354)	UNK
Wang et al.^15^	Chinese	Fasting hyperglycemia (5.5–8.0 mmoL/L) and glucose increment < 4.6 mmoL/L during 100 g OGTT	5.3 % (2/38)	0.4 % (2/501)
Siddiqui et al.^16^	Indian	Diagnosis of GDM with available DNA samples	0 % (0/51)	0 % (0/154)
Bitterman et al.^17^	Italian	Fasting hyperglycemia at the first visit in pregnancy ≥5.1 mmoL/L and negative GAD antibodies	38 % (8/21)	1.95 % (8/409)
Jiang et al.^18^	Chinese	Diagnosis of GDM	3.6 % (15/411)	3.6 % (15/411)

*GCK*-MODY, Maturity Onset Diabetes of the Young caused by *GCK* gene mutations; GDM, Gestational Diabetes Mellitus; UNK, Unknown; OGTT, Oral Glucose Tolerance Test; T2DM, Diabetes Mellitus of type-2; BMI, Body Mass Index; GAD, Glutamic Acid Decarboxylase.

## Materials and methods

### Study design and participants

This study retrospectively analyzed pregnant women in North China who received prenatal care or delivered at Beijing Jishuitan Hospital from April 1 to December 31, 2019. Clinical data and demographic data, such as age, ethnicity and parity, were extracted from electronic medical records using a predefined protocol. The study protocol was approved by the Ethics Committee of Beijing Jishuitan Hospital, with approval numbers 201805-17 and K2024–199-00. All procedures performed in this study were in compliance with the Declaration of Helsinki. Written informed consent was obtained from all participants or their legal guardians. All data used in this study were fully anonymized and de-identified prior to analysis. Personal identifiers (e.g., names, hospital ID numbers) were removed by the data custodians at Beijing Jishuitan Hospital before researchers accessed the dataset. Throughout the study, researchers had no access to information that could identify individual participants.

After pregnancy confirmation, participants underwent several tests, including fasting blood glucose during the first trimester (typically at 6–10 weeks), followed by routine prenatal examinations. A 75 g Oral Glucose Tolerance Test (OGTT) was conducted at 24–28 weeks of gestation. Gestational Diabetes Mellitus (GDM) was diagnosed using the International Association of Diabetes and Pregnancy Study Groups (IADPSG, 2010) criteria.^20^ GDM was diagnosed if one or more plasma glucose levels met or exceeded the following thresholds: fasting glucose ≥5.1 mmoL/L, 1-hour glucose ≥10.0 mmoL/L, or 2-hour glucose ≥8.5 mmoL/L. Participants who did not undergo the 75 g OGTT during pregnancy or were diagnosed with diabetes prior to pregnancy were excluded.

### Genetic analysis

Participants diagnosed with GDM with fasting blood glucose ≥5.1 mmoL/L underwent GCK gene sequencing and were designated as the GCK group. Sequencing was performed using residual blood samples from routine clinical tests, such as complete blood count with EDTA anticoagulant, without additional blood collection. Genomic DNA was extracted from whole blood samples, followed by amplification and sequencing as described previously.^15^ Sanger sequencing was conducted to analyze all exons, intron-exon boundaries, and promoter regions of the GCK gene using an ABI 3730XL DNA Analyzer (Applied Biosystems, Foster City, CA, USA).

Sequences were compared to the reference sequence (NM_000162.5) using the BLAT search tool (http://genome.ucsc.edu/cgi-bin/hgBlat) from the University of California, Santa Cruz (UCSC). The annotation tools utilized included the Genome Aggregation Database (gnomAD v4.1.0) and the 1000 Genomes (1000 G) database. Variants with a frequency ≤ 0.5 % were analyzed.

### Genetic variant classification and pathogenicity assessment

The clinical relevance of rare genetic variants was systematically evaluated through authoritative databases ClinVar (2025–02–01 release). For novel variants not previously documented, pathogenicity classification was conducted in strict accordance with the evidence-based framework established in the American College of Medical Genetics and Genomics (ACMG) 2015 guidelines. Computational prediction of coding region variant pathogenicity employed a multi-algorithm consensus approach, integrating PROVEAN and SIFT (http://provean.jcvi.org), PolyPhen-2 (http://genetics.bwh.harvard.edu/pph2), and MutationTaster (http://www.mutationtaster.org). Variants at non-coding regions were assessed using CADD (https://cadd.gs.washington.edu/score; v1.7), SpliceAI (https://spliceailookup.broadinstitute.org), and TraP-score (https://trap-score.org/; v 3.0).

### Statistical analysis

Stata software (version 16.0, StataCorp LP, USA) was used to analyze the variables. Continuous variables were presented as means ± Standard Deviation (SD), while categorical variables were reported as frequencies and percentages. The Student's *t*-test was used to compare normally distributed quantitative variables, and the Wilcoxon rank-sum (Mann-Whitney) test was used for non-normally distributed variables between two groups. A p-value of < 0.05 was considered statistically significant.

## Results

**Fig. 1:**
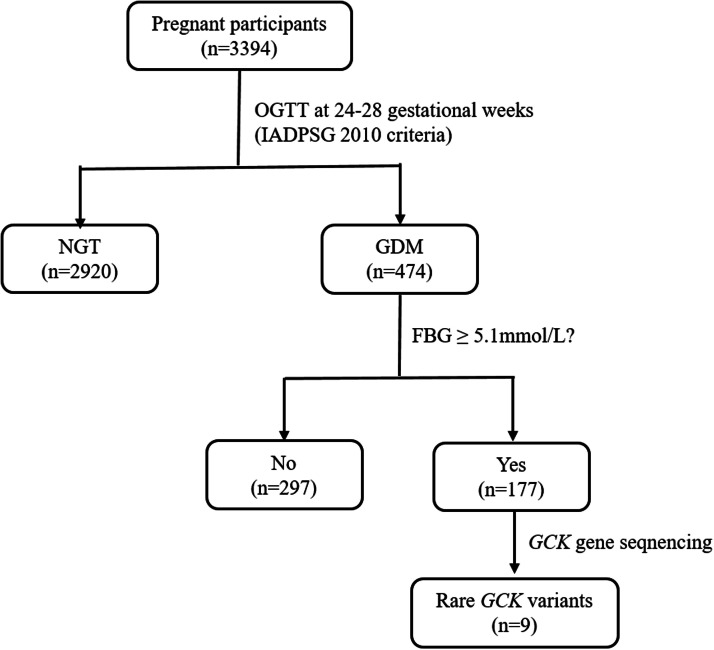
Algorithm of the study. OGTT, Oral Glucose Tolerance Test; IADPSG, International Association of Diabetes and Pregnancy Study Groups; NGT, Normal Glucose Tolerance; GDM, Gestational Diabetes Mellitus; FBG, Fasting Blood Glucose.

This study enrolled 3394 participants, of whom 96.2 % (3265/3394) were of Han Chinese ethnicity. Among the cohort, 474 individuals were diagnosed with GDM, and 177 underwent *GCK* gene sequencing, which identified nine carriers of rare GCK variants (Fig. 1). The overall GDM prevalence was 14.0 %. Comparative analysis revealed significant differences between GDM and Normal Glucose Tolerance (NGT) groups: GDM subjects were older (mean age 31.51 vs. 30.43 years; *p* < 0.001), exhibited elevated first-trimester fasting blood glucose (5.07 vs. 4.76 mmoL/L; *p* < 0.001), and showed higher glycosylated hemoglobin levels (5.26 % vs. 5.08 %; *p* < 0.001) (Table 2). Notably, the GCK group demonstrated more pronounced metabolic derangements compared to the NGT group, with significantly increased age (32.05 vs. 30.43 years; *p* < 0.001), elevated early-pregnancy fasting glucose (5.28 vs. 4.76 mmoL/L; *p* < 0.001), and higher HbA1c values (5.36 % vs. 5.08 %; *p* < 0.001).

**Table 2:** Clinical and anthropometric data of the participants.

	NGT group (*n* = 2920)	GDM group (*n* = 474)	p-value (vs. NGT)	GCK group (*n* = 177)	p-value (vs. NGT)
**Age of conception (yr)**	30.43 ± 3.26	31.51 ± 3.64	<0.001	32.05 ± 3.71	<0.001
**FBG in early pregnancy**^a^	4.76 ± 0.38	5.07 ± 0.58	<0.001	5.28 ± 0.68	<0.001
**HbA1c (%)**	5.08 ± 0.26	5.26 ± 0.34	<0.001	5.36 ± 0.41	<0.001
**FBG (mmoL/L)**^b^	4.26 ± 0.33	4.79 ± 0.64	<0.001	5.44 ± 0.47	<0.001
**1h-BG (mmoL/L)**	7.07 ± 1.37	9.75 ± 1.56	<0.001	9.52 ± 2.04	<0.001
**2h-BG (mmoL/L)**	6.15 ± 1.01	8.38 ± 1.53	<0.001	7.89 ± 1.86	<0.001
**Increment of BG (mmoL/L)**	1.89 ± 1.00	3.59 ± 1.68	<0.001	2.44 ± 1.68	<0.001

NGT, Normal Glucose Tolerance; GDM, Gestational Diabetes Mellitus; FBG, Fasting Blood Glucose; HbA1c, Glycated Hemoglobin; 1h-BG, 1-hour Blood Glucose during 75 g OGTT; 2h-BG, 2-hour Blood Glucose during 75 g OGTT; Increment of BG, difference between FBG and 2h-BG.

a Fasting blood glucose during the first trimester (typically at 6–10 gestational weeks).

b Fasting blood glucose at 75 g OGTT (typically at 24–28 gestational weeks).

Longitudinal analysis identified distinct temporal glucose patterns. Both NGT (4.76 vs. 4.26 mmoL/L; *p* < 0.001) and GDM (5.07 vs. 4.79 mmoL/L; *p* < 0.001) groups displayed significant reductions in fasting blood glucose levels from the first trimester to the second trimester, whereas the GCK group exhibited a paradoxical elevation (5.28 vs. 5.44 mmoL/L; *p* < 0.001). Furthermore, post-glucose challenge responses differed substantially, with the GDM group demonstrating exaggerated glycemic excursions relative to NGT controls.

Genetic sequencing of the GCK group revealed eight heterozygous rare variants among nine participants (Table 3), comprising: n.480+8245 C>T, c.−279 C>T (rs997877536), c.−29 C>A, c.−5 T>C (rs193922251), c.363+9 C>T (rs200985182), c.435C>G (p.Pro145Pro; rs773281783), c.863+52 G>A, and c.864-10 C>T (rs749737733) (RefSeq NM_000162.5, NP_000153.1). These variants included one exonic variant and seven non-coding region variants, with three novel variants not previously reported in population databases gnomAD and 1000 G. For previously reported rare variants, the ClinVar database classified their clinical significance as “conflicting classifications of pathogenicity”. Regarding the novel non-coding region variants identified, the authors conducted functional predictions using computational tools and applied the ACMG guidelines for pathogenicity classification, ultimately categorizing them as Variants of Uncertain Significance (VUS).

**Table 3:** Rare *GCK* gene variations (RefSeq NM_000162.5, NP_000153.1) identified in Chinese subjects with GDM.

Chromosome position (GRCh38/hg38)	HGVS Consequence	VEP Annotation	Clinical Significance^a^	dbSNP (155)	gnomAD-EAS^b^	PHRED	SpliceAI	TraPv3 score
7-44189446 G>A	n.480+8245 C>T	Intron	VUS (ACMG)	‒	‒	‒	‒	0.013
7-44189232 G>A	c.−279C>T	5′ UTR	Conflicting classifications of pathogenicity (ClinVar)	rs997877536	0.00009867	13.44	0.00	0.028
7-44188982 G>T	c.−29 C>A	5′ UTR	VUS (ACMG)	‒	‒	14.54	0.00	0.102
7-44188958 A>G	c.−5T>C	5′ UTR	Conflicting classifications of pathogenicity (ClinVar)	rs193922251	0.0001337	7.203	0.00	0.01
7-44152262 G>A	c.363+9C>T	Intron	Conflicting classifications of pathogenicity (ClinVar)	rs200985182	0	3.404	0.00	0.079
7-44151004 G>C	p.Pro145Pro	Synonymous	Conflicting classifications of pathogenicity (ClinVar)	rs773281783	0.0043	7.377	0.02	0.057
7-44147598 C>T	c.863+52 G>A	Intron	VUS (ACMG)	‒	‒	14.88	0.00	0.017
7-44146628 G>A	c.864-10 C>T	Intron	VUS (ACMG)	rs749737733	0.0005346	9.051	0.00	0.12

HGVS, Human Genome Variation Society; VEP, Variant Effect Predictor; dbSNP, Data Base Reference SNP; gnomAD-EAS, Allele frequency of the mutated base at this variant site in the gnomAD-East Asian population database; PHRED, Normalized CADD score (a score ≥20 suggests a deleterious variant); SpliceAI, Lower scores indicate less impact on splicing; TraPv3 score, TraP-score v3.0 Browser (scores > 0.174 indicate possible pathogenicity; scores > 0.289 suggest likely pathogenicity); VUS, Variant of Uncertain Significance; ACMG, American College of Medical Genetics and Genomics; UTR, Untranslated Region; -, Data not available.

a For novel variants, apply the ACMG (2015) guidelines to assess their pathogenicity; for previously reported variants, provide descriptions of clinical significance from the ClinVar database (2025-2-1 release).

b None of the variants were identified in the 1000 Genomes Project, while some exhibited very low allele frequencies in the gnomAD-EAS population.

The n.480+8245 C>T variant was identified in two unrelated GDM cases. Carriers of rare variants demonstrated comparable blood glucose levels and clinical manifestations to those observed in typical GDM patients, with their specific clinical characteristics detailed in Table 4. In an effort to assess the potential intergenerational impact, the authors conducted post-hoc telephone follow-up on the offspring of the nine rare GCK variant carriers, who were currently 5‒6 years old. Two families were lost to follow-up. Among the remaining seven, one child (born to carrier M3399, who herself has a family history of diabetes) underwent blood glucose testing, with results within the normal range. The parents of the other six offspring reported normal child development without overt signs of diabetes; however, they declined further blood glucose or genetic testing for their children.

**Table 4:** Clinical manifestations of carriers with rare GCK variants.

Subject	HGVS Consequence	Zygosity	Age (yr)	Gx (n)	Px (n)	HbA1c (%)	FBG (mmoL/L)	1h-BG (mmoL/L)	2h-BG (mmoL/L)	FH of DM	Prenatal Therapeutic Interventions	Pre-pregnancy BMI (kg/m^2^)	GWG (kg)	GA (weeks)	NBW (g)
M584	n.480+8245 C>T	Het	34	2	2	4.7	5.1	7.2	6.7	No	Diet	26.0	12.5	40+2	3650
M681^a^	n.480+8245 C>T	Het	31	1	1	5.3	5.3	5.8	7	No	Diet	‒	‒	‒	‒
M2480	c.−279C>T	Het	32	1	1	5.6	5.1	11	10.8	No	Detemir 7u	22.7	10	40+3	3050
M2286^a^	c.−29 C>A	Het	28	1	1	4.9	5.4	7.9	5.2	No	Diet	‒	‒	‒	‒
B363	c.−5T>C	Het	27	1	1	5.3	5.1	9.3	6.7	No	Diet	26.0	21	40+2	4000
M330	c.363+9C>T	Het	30	1	1	5.8	5.3	11.1	10.1	No	Detemir 8u	30.5	7	39+4	3700
M3399	p.Pro145Pro	Het	34	1	1	5.4	5.2	8	7.7	Yes	Diet	21.9	10	39+6	3400
M89	c.863+52 G>A	Het	31	3	1	5.5	6.8	13.9	11.4	No	Aspart 6-8-6u, Detemir 4u	26.3	8	39+5	3500
M2439	c.864-10 C>T	Het	28	1	1	5.8	5.7	10.6	7.7	No	RI 6-8-6U, NPH 6U	27.5	2	40+1	3800

HGVS, Human Genome Variation Society; Gx, Gravidity; Px, Parity; FBG, Fasting Blood Glucose During OGTT; 1h-BG, 1-hour Blood Glucose during 75 g OGTT; 2h-BG, 2-hour Blood Glucose during 75 g OGTT; FH of DM, family history of Diabetes Mellitus; GWG, Gestational Weight Gain; GA, Gestational Age; NBW, Neonatal Birth Weight; RI, Regular Insulin; NPH, Neutral Protamine Hagedorn; -, Data not available.

a The subject received prenatal check-ups at Beijing Jishuitan Hospital but did not deliver here.

## Discussion

This single-center study revealed that the prevalence of GDM among pregnant women in North China was 14.0 %. This result is consistent with other studies conducted in China and is higher than the prevalence of GDM observed among Caucasian women.^12^^,^^21^^,^^22^ Although studies suggest that different ethnic groups may have similar prevalence rates of *GCK*-MODY,^23^ the higher prevalence of GDM and the increased susceptibility to type 2 diabetes in Asian populations make it more challenging to screen for *GCK*-MODY among Asian GDM patients.^24^ Among the 477 GDM patients, 9 (1.9 %) carried rare *GCK* gene variants. It is crucial to note that all identified variants were classified as Variants of Uncertain Significance (VUS) or had conflicting interpretations of pathogenicity in ClinVar. Consequently, none of the carriers met the criteria for a definitive diagnosis of GCK-MODY, highlighting the rarity of confirmed monogenic diabetes cases and the current uncertainty regarding the clinical significance of these specific rare variants in the Chinese GDM cohort.

Despite harboring different mutations, patients with *GCK*-MODY exhibit similar glycemic profiles: fasting blood glucose levels ranging from 5.5 to 8.0 mmoL/L, a 2-hour OGTT increment < 4.6 mmoL/L, and HbA1c levels slightly above normal but typically ≤ 7.5 %.^25^, ^26^ Based on these glycemic characteristics, in conjunction with other indicators such as Body Mass Index (BMI) or family history of diabetes, previous studies have reported that the prevalence of *GCK*-MODY in screening populations ranges from 0 % to 80 %. Among these screening criteria, fasting blood glucose is the most commonly used and likely the most significant screening indicator in research.

Establishing an appropriate screening cutoff value remains a significant challenge.^19^ Additionally, the differences in blood glucose levels between pregnant and non-pregnant states raise concerns about the applicability of *GCK*-MODY glycemic characteristics during pregnancy. As demonstrated in the present study, the fasting blood glucose level in healthy pregnant women during the second trimester was slightly lower than that during the first trimester, with a difference of approximately 0.5 mmoL/L. This was attributed to the increased glucose consumption required for fetal growth and development. Given these considerations, a fasting blood glucose cutoff of ≥ 5.1 mmoL/L may be more suitable for screening *GCK*-MODY in patients with GDM.

This study screened the *GCK* gene in patients with GDM who had fasting blood glucose levels ≥5.1 mmoL/L, but no cases of *GCK*-MODY were definitively diagnosed. However, a previous study estimated the minimum prevalence of *GCK*-MODY among Chinese GDM patients to be approximately 0.4 % (2/511).^15^ Another study reported a prevalence of 3.6 % (15/411) among Chinese GDM patients.^18^ In the latter study, no specific genetic screening criteria were established, and *GCK* gene sequencing was performed on all patients diagnosed with GDM. The study suggested that Chakera's combined screening criteria (fasting blood glucose ≥ 5.5 mmoL/L and BMI < 25kg/m^2^)[12] resulted in the misdiagnosis of 33 % (5/15) of *GCK*-MODY cases, rendering them unsuitable for the Chinese population, and recommended universal *GCK* gene sequencing for all Chinese GDM patients. In contrast, this study did not sequence all GDM patients, and the GDM cohort exhibited a lower average fasting blood glucose level (4.79 vs. 5.0 mmoL/L),^18^ which likely reflects a greater proportion of negative cases. This may partly explain the lower prevalence observed in the present study.

Additionally, given the uneven economic development and high prevalence of GDM in China, the accessibility of comprehensive genetic testing and the affordability of medical care need to be fully considered.^27^ Using multiple blood glucose measurements for screening could be more cost-effective than comprehensive *GCK* gene testing. For example, screening criteria could involve fasting blood glucose levels of ≥ 5.1 mmoL/L in both the first and second trimesters of pregnancy, or combining this criterion with postpartum blood glucose levels of ≥ 5.5 mmoL/L. Anyway, further large-scale studies and more confirmed *GCK*-MODY cases are needed to validate this approach.

The findings of this study must be interpreted with a clear distinction: while the carrier rate for rare GCK VUS was 1.9 %, the prevalence of definitively diagnosed GCK-MODY was 0 %. Therefore, it cannot be concluded that the identified VUS contribute to GDM risk without functional validation. The primary conclusion is that the prevalence of confirmed, pathogenic GCK-MODY is exceptionally low in this cohort. This distinction underscores the genetic heterogeneity of GDM and suggests that monogenic forms attributable to GCK are a rare etiology in this Chinese population. The 1.9 % VUS carrier rate, however, represents an important area for future functional studies. This finding contrasts with studies of polygenic GDM risk conferred by common variants in genes such as HTR2B,^28^ highlighting the spectrum of genetic contributions to gestational dysglycemia. The present work, focused on monogenic screening, thus complements broader strategies for personalized prenatal care, which encompass comprehensive preconception counseling and the use of clinical risk scores for associated comorbidities.^29^^,^^30^

This study has several limitations. First, the lack of functional validation and family segregation studies precludes definitive conclusions regarding the pathogenicity of the identified rare GCK variants, and thus, their clinical significance remains uncertain. Second, as noted in the Results, the incomplete offspring follow-up data preclude any assessment of variant heritability or risk of early-onset dysglycemia, a limitation that mandates future familial studies. Future studies involving functional assays, family co-segregation analysis, and longitudinal metabolic assessments of offspring are essential to clarify the role and heritability of these variants.

## Conclusion

In summary, this study of 3394 pregnancies demonstrated that *GCK*-MODY is rare among Chinese GDM patients. Despite the challenges of screening for *GCK*-MODY among GDM patients, pregnancy offers a critical opportunity to assess blood glucose levels and identify *GCK*-MODY in young women. Further research is needed, particularly basic studies on the pathogenicity of rare variants.

## Funding

This research was funded by the Youth Backbone Individual Project of Beijing Municipal Talent Development Funding (2017000021469G60), Beijing Jishuitan Hospital Nova Program (XKXX202109) and Beijing Jishuitan Hospital Elite Young Scholar Program (XKGG202119).

## Data availability

The datasets generated and/or analyzed during the current study are available from the corresponding author upon reasonable request.

## CRediT authorship contribution statement

**Zhixin Wang:** Conceptualization, Investigation, Writing – original draft. **Lili Huo:** Supervision, Investigation. **Ling Lan:** Investigation. **Yongzeng Chen:** Formal analysis. **Qingyao Zuo:** Investigation. **Wei Deng:** Supervision, Investigation.

## Declaration of competing interest

The authors declare that they have no known competing financial interests or personal relationships that could have appeared to influence the work reported in this paper.
